# Complications and Outcomes of the Internal Fixation of Non-displaced Femoral Neck Fracture in Old Patients: A Two-Year Follow-Up

**DOI:** 10.7759/cureus.41391

**Published:** 2023-07-05

**Authors:** Ahmad G Abdallatif, Anirudh Sharma, Tariq Mahmood, Nadim Aslam

**Affiliations:** 1 Trauma and Orthopaedics, Worcester Royal Hospital, Worcester, GBR; 2 Trauma and Orthopaedics, Hinchingbrooke Hospital, Cambridgeshire, GBR; 3 Trauma and Orthopaedics, Worcestershire Acute Trust, Worcester, GBR

**Keywords:** femoral neck fracture, avascular necrosis (avn), non-union, cannulated screw, dynamic hip screw (dhs)

## Abstract

Background

Fractures of the proximal femur are amongst the most common injuries in the elderly population. While there is a clear consensus regarding the management of displaced femoral neck fractures, the management of non-displaced fractures is less clear. Both fixation and arthroplasty are valid treatment options. Internal fixation is a less invasive procedure, but it carries the risks of non-union and avascular necrosis (AVN) of the femoral head. The literature describes varying complication rates associated with these risks. We aim to describe a series of elderly patients above the age of 65 years with non-displaced fractures of the femoral neck who were treated with internal fixation. Our objectives are to determine the union rate and complications in this group and to elucidate the factors that influence these outcomes.

Methods

We conducted a retrospective review of all patients aged 65 years and older who presented with femoral neck fractures at our level 1 trauma unit between 2018 and 2020. Fractures were classified using the Garden classification system, and only those with Garden 1 or 2 fractures (non-displaced) were included. Preoperative radiographs or intraoperative fluoroscopy images were used to classify fractures using the Pauwels classification. Serial postoperative radiographs and clinical notes (up to 24 months postoperatively) were reviewed to assess the union rate and the development of complications.

Both non-union and AVN were analysed for their associations with age, sex, Pauwels grade and comorbidities. A subgroup analysis of the complications was performed to elucidate their association with age groups (<80 and >80 years) and types of fixations (dynamic hip screws {DHS} and cannulated screws).

Results

A total of 148 patients, consisting of 60 males and 88 females, were included in the analysis. The patients had a mean age of 78.5 years (ranging from 65 to 98 years). The union rate without any degree of AVN was 90.7%, with six non-unions (4.05%) and six patients experiencing AVN (4.05%). No difference in outcome was detected between the two groups based on age.

High (type 2 or 3) Pauwels grade (p = 0.05) and treatment with cannulated screws (p = 0.02) were indicated as significant factors for non-union. All patients who developed AVN were noted to have a comorbidity that is known to predispose them to AVN.

Conclusion

Our series shows a union rate of 90.7%, which is comparable to the union rates reported in other published literature. Our results suggest that age does not independently influence the outcome of fixation for these fractures. We conclude that fractures with vertical orientation (Pauwels grade 2 or 3), when treated with cannulated screws, are more likely to result in non-union. AVN is the second most common complication after non-union, which is also associated with other risk factors for AVN.

## Introduction

An increase in the average lifespan has led to an increase in the number of femoral neck fractures, which are one of the most common causes of death in the age group 65 years and older due to postoperative complications, mainly chest infection and kidney failure. The overarching aim of treating fractures such as these is to achieve early pain-free mobility through a single optimal procedure. The management method for this type of fracture depends on several factors, including the patient’s age, displacement, comorbidity, pre-fracture activity and posterior angulation at the fracture site [1].

With a displaced fracture, hemiarthroplasty and total arthroplasty are the preferred choices for elderly patients according to the National Institute for Health and Care Excellence (NICE) guidelines. These procedures have shown significant improvement in function and pain management, with lower rates of complications and reoperation compared to internal fixation [2,3]. However, the treatment of non-displaced femoral neck fractures remains unclear. The Pauwels and Garden classifications are commonly used to classify fractures of the femoral neck based on the degree of displacement at the fracture site. Non-displaced fractures include Garden 1 (incomplete and non-displaced) and Garden 2 (complete and non-displaced) fractures. The treatment options in such cases include either fixation or arthroplasty [4-6].

With Garden 1 and 2 femoral neck fractures, internal fixation has traditionally been the preferred method of treatment, regardless of age. Satisfactory outcomes have been achieved with advantages such as shorter operative time, reduced blood loss and the preservation of the native joint. The internal fixation of a stable femoral neck fracture shows superior functional outcomes and a lower morbidity rate compared to conservative treatment [7].

Recently, some studies have shown a high rate of reoperation in the management of non-displaced fractures in the elderly population. This can be attributed to various factors such as implant failure (non-union), avascular necrosis (AVN) or collapse at the fracture site, leading to a decrease in abductor moment [8,9]. Given these complications, there have been a number of systematic reviews and meta-analyses comparing the outcomes between hemiarthroplasty and fixation for non-displaced femoral neck fractures [10]. These studies have unanimously shown a lower rate of complications and reoperations with hemiarthroplasty [10].

However, fixation continues to have a significant value in the treatment of these fractures, with excellent outcomes reported in several studies [11,12]. There has been an interest in elucidating the factors that determine the success or failure of fixing such fractures. The posterior tilt angle is a factor that has been studied in the literature. It has been found that an angle of more than 20 degrees is associated with poor outcomes in fixation [13]. The Pauwels classification similarly influences fixation, with vertically oriented fracture lines being relatively unstable compared to horizontally oriented fractures [14]. The method of fixation can also vary, with multiple cannulated screws (MCS) or a dynamic hip screw (DHS) being described to treat such fractures [15].

We aimed to review a series of elderly patients with non-displaced neck fractures treated at our institution using two methods of internal fixation. Our goal was to determine the factors, such as age, the type of fixation and the type of fracture, that influenced the outcome. Knowledge of these factors would help surgeons predict which fractures would have a good outcome with fixation and, conversely, perform arthroplasty for those predisposed to failure.

## Materials and methods

All patients with fractures of the femoral neck who presented to our institution between January 2018 and September 2020 were retrospectively reviewed. The patients who were younger than 65 years of age at the time of the injury were excluded. The Garden classification was used to categorise the fractures. Fractures classified as Garden 1 and Garden 2 were included in the analysis, while those classified as Garden 3 or Garden 4 were excluded.

**Figure 1:**
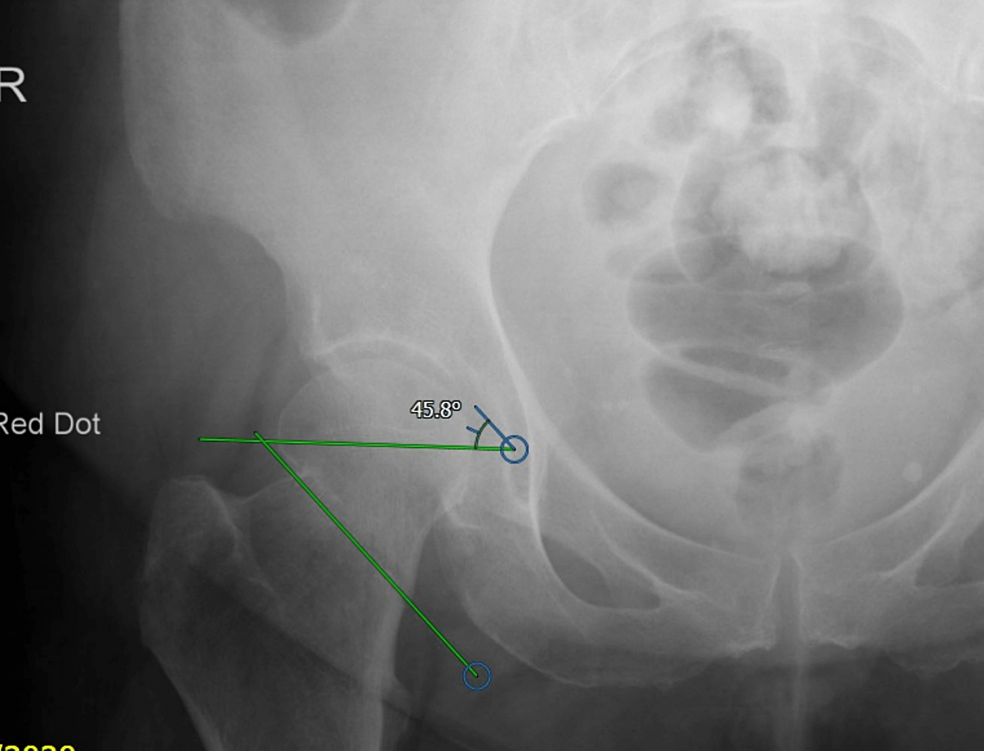
AP X-ray of the hip with NOF fracture (Pauwels 2) AP, anteroposterior; NOF, neck of femur

**Figure 2:**
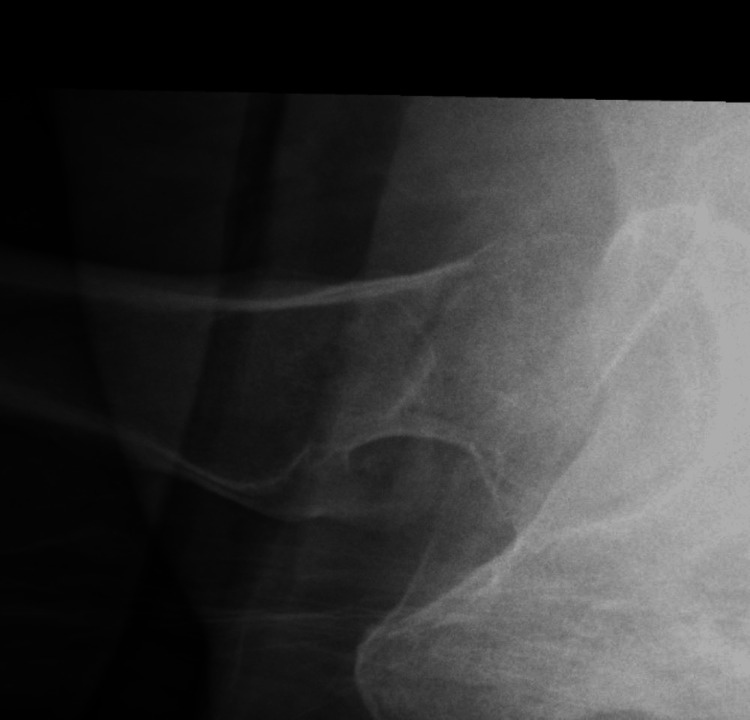
Lateral-view X-ray of the hip with NOF fracture NOF: neck of femur

**Figure 3:**
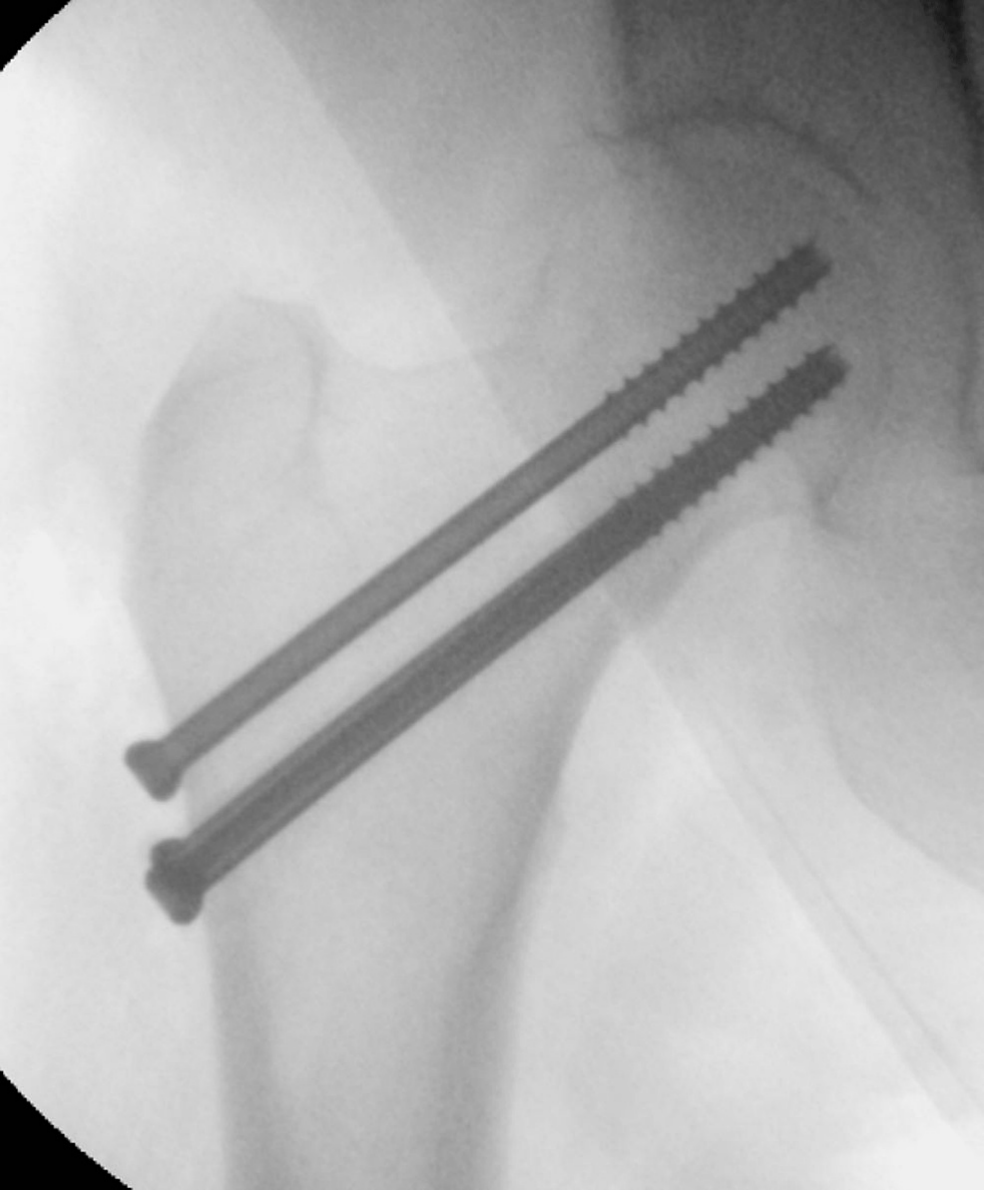
AP intraoperative image of the fracture fixed with MCS AP, anteroposterior; MCS, multiple cannulated screws

**Figure 4:**
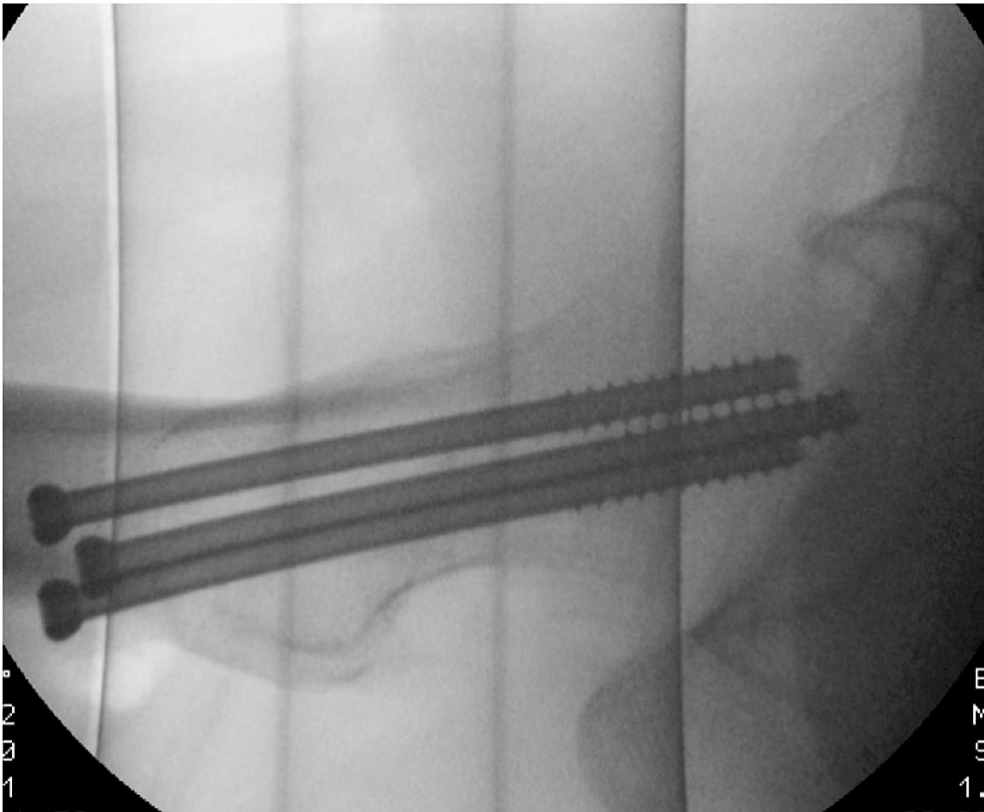
Intraoperative lateral-view image

The preoperative parameters that were noted included age, gender, the side of injury and medical comorbidities (if any). Preoperative radiographic parameters included the measurement of the Pauwels classification of the fracture using the institution’s picture archiving and communication system (PACS) digitally (Figures 1-2). If the preoperative films were unclear, the intraoperative fluoroscopy images were used for measurements. The method of fixation used was noted, which was either multiple cannulated screws (MCS) or a dynamic hip screw (DHS) (Figures 3-4).

Postoperative radiographic records were followed up sequentially to assess union, with a minimum follow-up period of 24 months. Those who did not have radiological records until 24 months or those who died prior to this period were excluded from the analysis. Clinical notes from the outpatient department were reviewed to identify the development of any additional complications.

**Figure 5:**
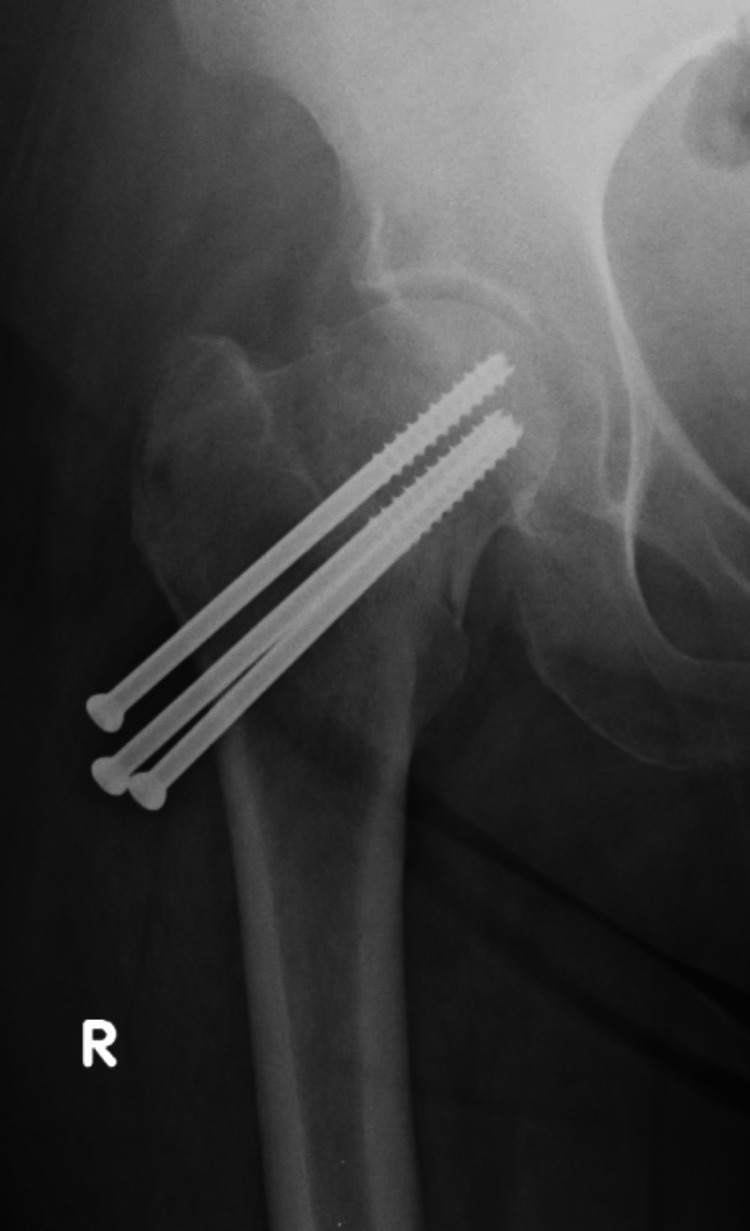
AP X-ray shows non-union with fracture collapse AP: anteroposterior

**Figure 6:**
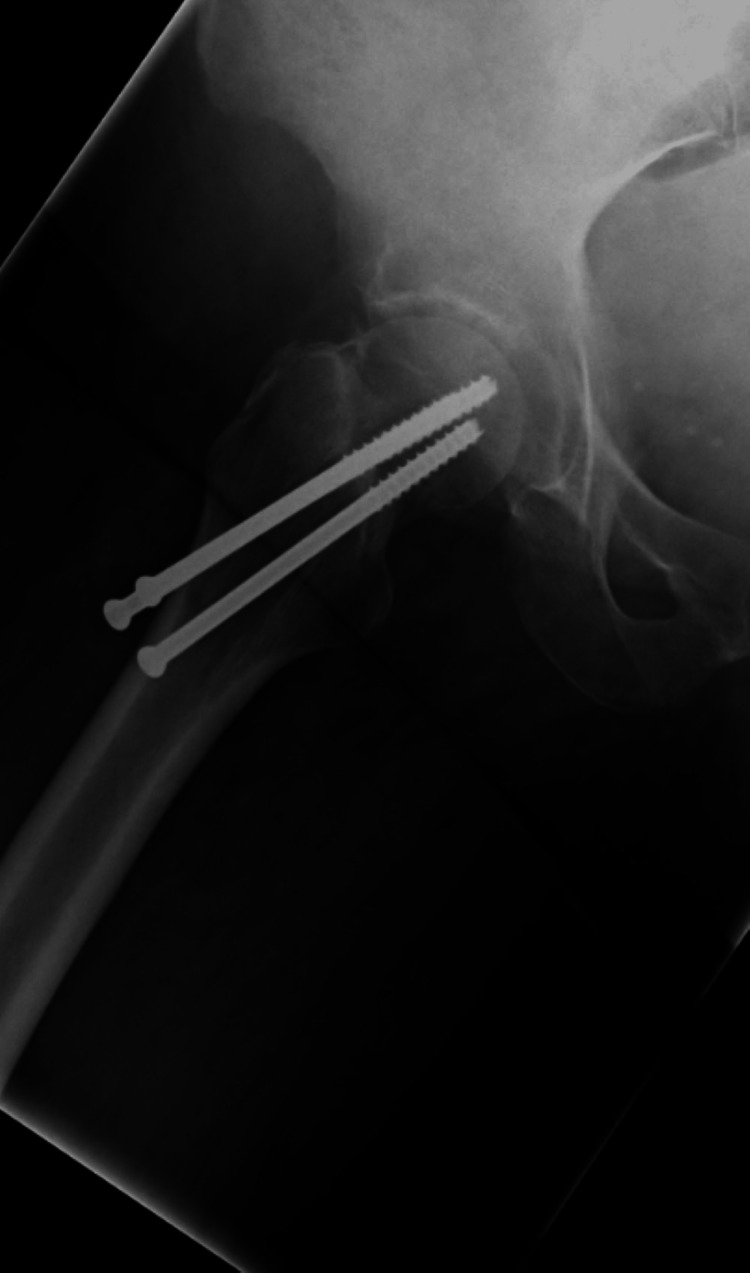
Lateral-view X-ray of fracture non-union

**Figure 7:**
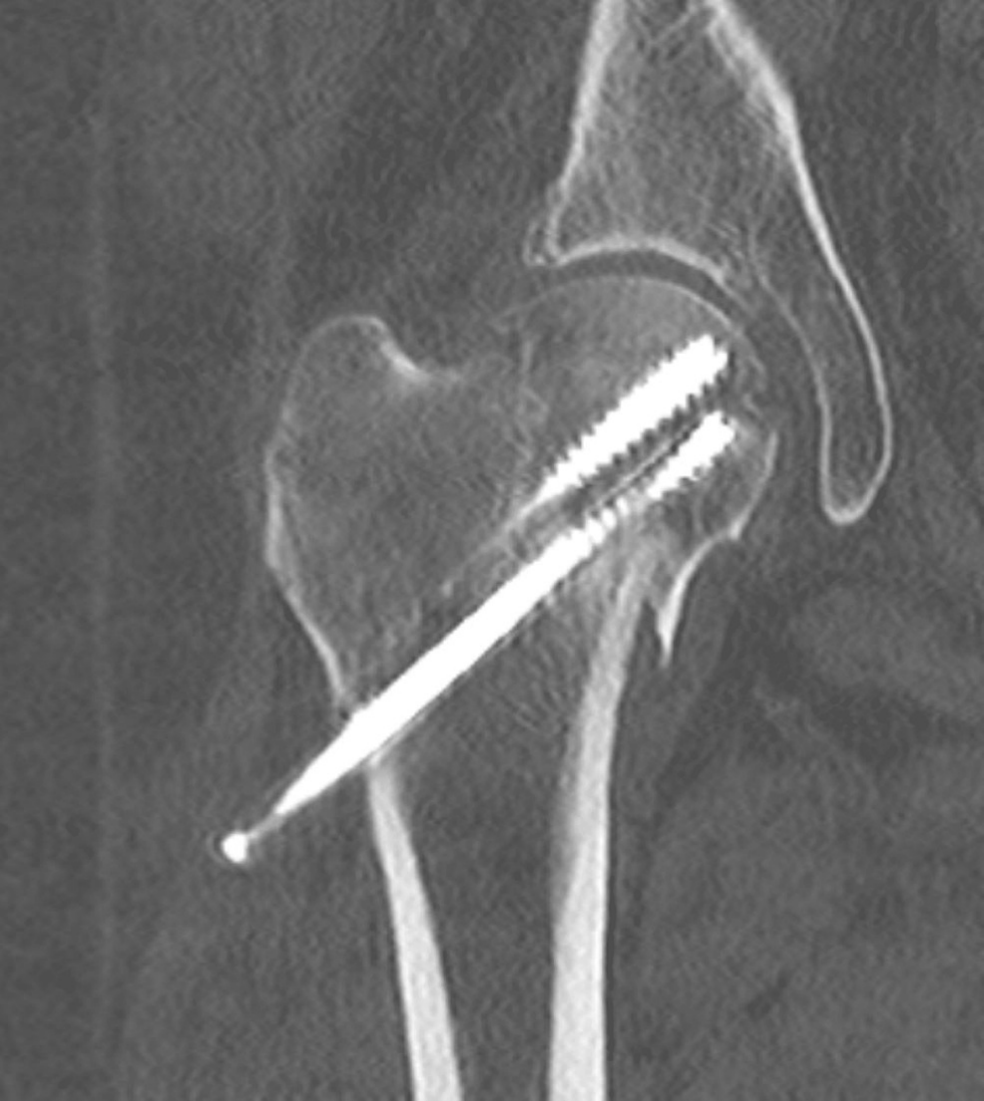
CT scan confirmed non-union at the fracture site

We assessed the rate of non-union (Figures 5-7), the rate of AVN development and the revision rate of this series. We evaluated the patients with non-union and AVN to determine the factors associated with their development, including age, sex, side, Pauwels grade and comorbidities. We performed a subgroup analysis based on age (<80 years or >80 years) and the type of fixation (MCS versus DHS).

## Results

Chi-square tests have been used to detect the relationships between age group, posterior tilt angle, fracture orientation, the method of fixation and reoperation rate due to the failure of fixation or AVN.

After applying the exclusion criteria, we included 165 cases of Garden 1 or 2 fractures in our series. These cases were the patients who presented to our institution between January 2018 and September 2020. Of these, 17 patients had died within the two-year postoperative period due to unrelated medical causes and were therefore excluded from the study. This resulted in a total of 148 patients being included in the final analysis.

These included 60 males and 88 females, with a mean age of 78.5 years (range: 65-98 years). A total of 80 patients underwent fixation with DHS, whereas 68 patients had a fixation with MCS. According to the Pauwels classification, 111 patients had a Pauwels 1 fracture, while 37 patients had Pauwels 2 or 3 fractures.

Overall, 136 out of the 148 patients (90.7%) achieved a successful union without any complications during the two-year follow-up period. Of the 12 patients who developed complications, six patients (4.05%) were found to have non-union, and six patients developed AVN of the femoral head (4.05%). Of these, 10 required revision surgery: eight underwent total hip arthroplasty, whereas two had the removal of metalwork (Table 1).

**Table 1:** Complications associated with two types of fixation AVN, avascular necrosis; NS, not significant

Patient age and complication	All patients (n = 148)	Group		Test of significance (p value)
		Dynamic hip screw fixation (n = 80)	Cannulated screw fixation (n = 68)	
Age (years): minimum to maximum	65.00-98.00	65.00-98.00	65.00-98.00	
Age: mean ± standard deviation	78.510 ± 8.93	79.56 ± 9.47	77.28 ± 8.16	
Complications: AVN	6 (4.05%)	1 (1.25%)	5 (7.35%)	P = 0.050 (NS)
Complication: non-healing	6 (4.05%)	1 (1.25%)	5 (7.35%)	P = 0.050 (NS)

A subgroup analysis of the patients with complications is shown in Table 2. There was no significant difference in complication rates between the two age groups (p = 0.4) or between genders (p = 1.2). Of the six patients who developed non-union, five (83.3%) received treatment with MCS, and this was found to be statistically significant when compared to DHS (p = 0.05). Similarly, five patients (83.3%) who developed AVN of the femoral head were treated with MCS instead of DHS (p = 0.05).

**Table 2:** A subgroup analysis of the patients with complications AVN, avascular necrosis; NS, not significant

Complication	Age group		P value	Pauwels 2 and 3	P value
	Below 80 years (n = 74)	80 years or above (n = 74)			
AVN	4 (5.41%)	2 (2.70%)	P = 0.406 (NS)	2	0.23
Non-healing	4 (5.41%)	2 (2.70%)	P = 0.406 (NS)	5	0.05

When comparing the complication rates based on Pauwels grade, seven out of 37 patients with Pauwels 2 or 3 fractures developed a complication, whereas only five out of 111 patients with Pauwels 1 fractures developed a complication (p = 0.032). In patients with non-union, five out of six (83.3%) had Pauwels 2 or 3 fracture, which was statistically significant (p = 0.05). However, no significant association was found between Pauwels grade and AVN (p = 0.23).

A combination of high Pauwels grade fractures treated with cannulated screws was found to have a significantly higher non-union rate (p = 0.001) compared to Pauwels 1 fractures treated with DHS.

Five patients who had AVN had risk factors for AVN. One patient had a history of previous radiation for the management of breast cancer, and two patients had a history of chronic steroid use for the management of rheumatoid arthritis. One patient has a history of heavy smoking and alcohol abuse, while another patient has a history of chemotherapy. Five patients underwent fixation with cannulated screws, while one patient received a DHS, which is a significant difference (Table 3).

**Table 3:** Relation of AVN incidence and the presence of other risk factors AVN: avascular necrosis

Patient number	Side	Age	Sex	Risk factors for AVN	Pauwels classification	Reoperation	Type of fixation
AVN 1	Right	92	Female	No risk factor	1	Removal of implant	Multiple cannulated screws (MCS)
AVN 2	Left	82	Male	Radiotherapy	1	None	Dynamic hip screw (DHS)
AVN 3	Right	67	Male	Chronic steroid treatment	1	None	Multiple cannulated screws (MCS)
AVN 4	Right	65	Female	Alcohol abuse	2	Total hip replacement (THR)	Multiple cannulated screws (MCS)
AVN 5	Left	65	Female	Chronic steroid treatment	1	Total hip replacement (THR)	Multiple cannulated screws (MCS)
AVN 6	Left	75	Male	Chemotherapy	1	Total hip replacement (THR)	Multiple cannulated screws (MCS)

## Discussion

The results of our series demonstrate that non-union after the fixation of femoral neck fractures appears to be independent of age but is associated with a higher Pauwels grade (2 or 3) and the use of cannulated screws. Also, 83.3% of the patients who developed AVN had an associated comorbidity known to predispose them to AVN.

The rate of overall non-union and AVN in our series is similar to that reported in previous studies [13,16]. A study conducted in the United Kingdom involving 375 patients who underwent internal fixation for non-displaced femoral neck fractures reported a non-union rate of 6.4% and an AVN rate of 4%. These findings are consistent with our own series [16]. There have been numerous studies conducted to determine the factors that influence outcomes following the fixation of femoral neck fractures. However, the results of many studies have been contradictory, and as a result, the evidence remains unclear. While some studies have suggested that age is a determinant of non-union (Parker et al.) [13], others (Nilsson et al. [17] and Yang et al. [18]) have refuted this. Similarly, while the posterior tilt angle of the fracture has been shown to be predictive of failure in studies such as those of Kalsbeek et al. [19] and Clement et al. [20], a study with a five-year follow-up suggested that this was not predictive of failure (Lapidus et al.) [21].

The importance of the fixation method used to treat these fractures has also been studied. Yih-Shiunn et al. compared the use of MCS to DHS to treat non-displaced femoral neck fractures and found a significant difference in the overall success rate. DHS was found to be superior to MCS, with a success rate of 94.1% [22]. However, Cullen et al. [23] found no difference in complication rates or revisions when comparing the use of MCS to DHS in non-displaced fractures. A systematic review found that a DHS is superior to cannulated screws in terms of postoperative complications and has better union times compared to an MCS [24]. Our results show that cannulated screw fixation, when associated with a high Pauwels grade, leads to non-union.

The Pauwels classification was described in 1935 to help predict fractures that are at risk of displacement and non-union. A number of studies have since shown that it is a poor predictor of non-union [25]. However, others, such as the study by Zhu et al. [26], have found it useful in predicting reoperation after initial fixation. The Dutch Surgical Society guidelines for the treatment of femoral neck fractures are based on Pauwels grade of the fracture [27]. They recommend the use of a sliding hip screw instead of cannulated screws for Pauwels 3 fractures. Our findings from this series further strengthen this recommendation, showing that cannulated screws are not effective for treating such fractures.

Our results showed that all patients who developed AVN had medical comorbidities known to predispose them to the condition (Table 3). A recent meta-analysis has shown that displacement, as per the Garden classification, is the only factor predisposing to this condition. Age, gender, time to operation and the mode of reduction have no effect [24]. We believe that the presence of comorbidities had a greater effect on the development of AVN in our series than the use of cannulated screws.

This paper has some limitations, including a short follow-up period of two years. It also does not include an objective assessment of hip joint function after internal fixation, such as the Oxford hip score. It did not check the quality of fracture reduction in postoperative X-ray, which is one of main factors of fracture union. The paper compared two types of internal fixations, MCS and DHS, but did not include a comparison with arthroplasty alongside internal fixation. Further study to compare two methods of treatment, fixation versus arthroplasty, is still highly required.

## Conclusions

Our series shows a union rate of 90.7%, which is comparable to the union rates reported in other published literature. Age does not independently predict the outcome of fixation in non-displaced femoral neck fractures in the elderly (>65 years) population. A combination of factors, namely, high Pauwels grade fractures treated with cannulated screws (rather than DHS), is highly predictive of non-union. Avascular necrosis incidence is associated with comorbidities known to predispose to this condition.
